# Genotoxic and oxidative effect of duloxetine on mouse brain and liver tissues

**DOI:** 10.1038/s41598-021-86366-0

**Published:** 2021-03-25

**Authors:** Isela Álvarez-González, Scarlett Camacho-Cantera, Patricia Gómez-González, Michael J. Rendón Barrón, José A. Morales-González, Eduardo O. Madrigal-Santillán, Rogelio Paniagua-Pérez, Eduardo Madrigal-Bujaidar

**Affiliations:** 1 Laboratorio de Genética, Instituto Politécnico Nacional, Escuela Nacional de Ciencias Biológicas, Av. Wilfrido Massieu s/n. Zacatenco, Ciudad de México, 07738 México; 2 Laboratorio de Medicina de La Conservación, Instituto Politécnico Nacional, Escuela Superior de Medicina, Plan de San Luis Y Díaz Mirón S/N, Casco de Santo Tomás, Ciudad de México, 11340 México; 3 Servicio de Bioquímica, Instituto Nacional de Rehabilitación, Av. México-Xochimilco 289, Ciudad de México, 14389 México

**Keywords:** Drug safety, Toxicology

## Abstract

We evaluated the duloxetine DNA damaging capacity utilizing the comet assay applied to mouse brain and liver cells, as well as its DNA, lipid, protein, and nitric oxide oxidative potential in the same cells. A kinetic time/dose strategy showed the effect of 2, 20, and 200 mg/kg of the drug administered intraperitoneally once in comparison with a control and a methyl methanesulfonate group. Each parameter was evaluated at 3, 9, 15, and 21 h postadministration in five mice per group, except for the DNA oxidation that was examined only at 9 h postadministration. Results showed a significant DNA damage mainly at 9 h postexposure in both organs. In the brain, with 20 and 200 mg/kg we found 50 and 80% increase over the control group (*p* ≤ 0.05), in the liver, the increase of 2, 20, and 200 mg/kg of duloxetine was 50, 80, and 135% in comparison with the control level (*p* ≤ 0.05). DNA, lipid, protein and nitric oxide oxidation increase was also observed in both organs. Our data established the DNA damaging capacity of duloxetine even with a dose from the therapeutic range (2 mg/kg), and suggest that this effect can be related with its oxidative potential.

## Introduction

A major depressive disorder is one of the most common and debilitating mental problem worldwide. The disease is characterized by impairments in cognition, emotional regulation, memory, and motoric function, motivation, and neurovegetative symptoms; in addition to these primary effects, the disorder can also cause several secondary disabilities which may represent a high economic burden for the involved family and the government^1^. Pharmacotherapy plays an important role in the disease treatment although there is no consensus about which drug can be the most useful as a first option, considering the possibility of its long-term use and the patient´s clinical variability^2^.

A study about the relative efficacy, acceptability, and tolerability of antidepressants concluded that one of the recommended drugs for depression is duloxetine^3^; besides, the medicament is also used against other mental problems, as well as inflammation and pain. Duloxetine is a serotonin and norepinephrine uptake inhibitor, that has a low affinity for most 5-HT subtypes, and muscarinic, histamine H1, alpha1-adrenergic, alpha2-adrenergic, and dopamine D2 receptors^4^.

Toxicological studies of the drug have shown similar moderate collateral effects as observed in most antidepressants, mainly including the gastrointestinal and nervous systems, and few aggressive damage in specific organs, such as in the liver^5^.

Concerning the genotoxic field and related areas, few studies have been published in in vitro and in vivo assays. Di Poi et al.^6^ detected embryotoxicity induced by the examined medication in the oyster *Crassostrea gigas*, Lassen et al.^7^ observed an increase in the rate of outcomes with major congenital malformations during the first trimester of pregnancy in women under treatment; from 668 infants the authors found 16 with malformations and estimated relative risk of 0.80. Concerning genotoxic and carcinogenic studies, Brambilla et al.^8^, summarized the effect of the antidepressant in various in vitro and in vivo assays with negative results although the tested doses were not provided; however, the authors reported a positive carcinogenic effect in the rat liver, Respect to its in vivo genotoxic potential, a report published by Madrigal-Bujaidar et al.^9^ evaluated the capacity of the drug to induce micronuclei in mouse blood cells, and found a moderate effect of the antidepressant in both, an acute and a subchronic assay; besides, by examining the number of sister chromatid exchanges in mouse bone marrow, the same authors again demonstrated a moderate but significant increase of this parameter^10^. Respect to the application of the comet assay, a previous report revealed no damage by the antidepressant in the mouse brain and blood cells^11^. However, considering that observations were made 24 h after the drug exposure, we feel pertinent to re-apply the same assay using a different strategy; therefore, a first objective of the present report was to examine the drug´s capacity to affect the DNA using the comet assay in the mouse brain and hepatic cells, but using a kinetic-time design in an attempt to detect the drug’s effect at four different time-points after the mouse exposure. Besides, in light of the previously reported chromosomal damage by duloxetine, and because of the presence of potentially oxidant chemical groups in the molecule, such as the naphthyl ring, and the potential oxidative effect during its metabolism due to the formation of epoxides, a second objective of the present study was to also perform a kinetic-time study on the oxidative capacity of the medication regarding DNA, lipids, proteins, and nitric oxide.

## Materials and methods

### Chemicals and animals

Duloxetine hydrochloride was obtained as the usually prescribed antidepressant (Cymbalta, Eli Lilly & Co., Mexico), CAS number 136434-34-9, molecular formula C_18_H_19_NOS^12^. The following substances were obtained from Sigma Chemicals (St Louis Mo. USA): triton X-100, dimethyl sulfoxide (DMSO), methyl methanesulfonate (MMS), sodium chloride, tris, normal melting point agarose (NMPA), low melting point agarose (LMPA), calcium and magnesium free phosphate-buffered saline (PBS), ethydium bromide (EB), trypan blue solution, N-lauroyl-sarcosine (sodium salt), HEPES, bovine serum albumin (BSA), Bradford reagent, thiobarbituric acid (TBA), trichloroacetic acid **(**TCA), 2,4-dinitrophenylhydrazine (DNPH), guanidine, sulfanilamide, N-1-(naftil) etilendiamine dichloride, and the enzyme formamidopyrimidine-DNA glycosylase (FPG)**.** Besides, potassium hydroxide, potassium chloride, sodium hydroxide, EDTA, ethanol, ethyl acetate, formic acid, methanol, and hydrochloric acid (HCl) were purchased from Baker (Phillipsburg NJ, USA).

For the assay, we used 100 male mice (ICR) with a mean weight of 23 g (Harlan Laboratory, Mexico City). Five mice per cage and 20 per experimental group were placed in polycarbonate cages at 24 °C, 12 h dark–light cycles, 50% relative humidity, and with free access to water and food (Rodent Chow 5001, Purina). The experiment was approved by the Bioethics Committee of the Hidalgo State Autonomous University (Mexico) and was started after a week of animal stabilization in the Genetics animal facility, according to the previously indicated conditions. Besides, we confirm that all used methods in the present research were performed according to the recommended international guidelines and regulations, as reported in ARRIVE guidelines, published in https://arriveguidelines.org^13^.

For the standard comet assay we had the following groups of animals with twenty mice each: a control group intragastrically administered purified water, a positive control group intraperitoneally administered 150 mg/kg of methyl methanesulfonate, and three groups intragastrically administered duloxetine in the doses of 2, 20, and 200 mg/kg. The highest dose corresponded to 70% of the DL_50_ previously obtained in our laboratory (282 mg/kg by the intragastric route), and the low dose corresponded to the high dose range recommended for daily therapeutic use in humans. The same mentioned chemicals and doses of duloxetine were used in the other oxidative tests applied in the present work. Observations for each genotoxic and oxidative parameters were made at 3, 9, 15, and 21 h post-administration, except for the comet assay plus FPG that was made at 9 h, only.

### Comet assay: Standard technique

Each mouse was dissected to obtain the brain and the liver. Concerning the brain, about 4 mm of the tissue were deposited in 250 ml of PBS, repeatedly hit with a syringe plunger to finally place 40 ml of the cell suspension on ice, while the liver was disaggregated with scissors, the lumps eliminated and the cells also placed in cold PBS. The comet assay procedure was based on published guidelines on the method^14^. We used about 10 000 cells/ml in each tested sample with the viability of more than 80% according to the trypan blue staining method.

We used fully frosted slides coated with three layers of agarose: initially, 120 µl of 1% NMPA made in PBS were placed in a coverslip, left to solidify for 4 min at 4 °C and placed in a slide, then, on top of such layer of agar we added a second layer constituted by 75 µl of 1% LMPA made in PBS, plus 20 µl of the cell suspension (brain or liver), and finally, the last layer constituted by 75 µl of 1% LMPA was added. Three slides per treatment/exposure time were made, protected from light, and placed for 24 h in the lysis solution constituted by NaCl 2.5 M, EDTA 100 mM, tris 10 mM, sodium sarcosinate 1%, plus triton X-100 1% and DMSO 10%, pH 10. Slides were then placed in an electrophoresis chamber containing NaOH 300 mM, plus EDTA 1 mM at pH > 13 for 20 min before carrying out the electrophoresis at 25v, 250 mA, and pH > 13 for 20 min. After this step, cells were washed with tris (0.4 M, pH 7.5) for 5 min, and each slide was stained with EB (25 µg/ml). The comet tail length/nucleus diameter index was analyzed in 100 nucleoids per individual/treatment/ time utilizing an epifluorescent microscope (Axioscope, Carl Zeiss) equipped with emission and excitation filters of 488 and 565 nm, respectively. The microscope was adapted to an image analyzer Image-Pro Plus (Media Cybernetics).

The statistical analysis of the obtained results was made with the ANOVA test followed by the Student–Newman–Keuls test, using the Program SigmaStat version 3.5.

### Comet assay: With the inclusion of the FPG enzyme

To evaluate the capacity of duloxetine to oxidize the DNA molecule we applied the comet assay plus the addition of the FPG enzyme. Parallel slides initially prepared for the previous assay were used. However, in this case, after cells passed through the lysis solution, they were washed three times, 5 min each, with the enzyme buffer constituted by HEPES 40 mM, KCl 0.1 M, EDTA 0.5 mM, and albumin bovine serum 0.2 mg/ml, at pH 8.0. The obtained enzyme had 10 µg of FPG. These were diluted in 2 ml of the buffer solution to produce a stock solution of 5 µg/ml, then, 10 µl of such solution were added to 40 ml of buffer. In this form we obtained an FPG final concentration of 1 µg/ml. We added the 50 µl the final solution to each slide that was covered it with a cover slide and placed in a humid chamber for 45 min at 37 °C. Slides were then placed at 4 °C for 5 min, the coverslips were removed, and the DNA denatured with a solution of NaOH 300 mM, plus EDTA 1 mM, at pH 13 for 40 min; finally, the electrophoresis was carried out at 25 v, 300 mA, and pH > 13 for 30 min^15^. After this step, the procedure, scoring, and statistical analysis were made as described above for the standard technique.

### Total protein determination

For this determination, we followed the method described by Bradford^16^. Initially, the tissues were homogenized in PBS (1:10), then, 100 μl of homogenate from each tissue was centrifuged at 9000 rpm for 10 min, and 10 µl of the obtained supernatant was mixed with 90 μl of deionized water and 2.5 ml of Bradford’s reagent, after which the mix was agitated for 5 min. Samples were spectrophotometrically read at 595 nm against a blank made with 100 ul of deionized water plus 2.5 ml of Bradford´s reagent. The results were interpolated in a bovine serum albumin standard curve (0.1 to 1.0 mg/ml) and expressed as mg protein/g tissue.

### Determination of lipid peroxidation

For this determination, we used the method of Buege and Aust^17^, that register the concentration of malondialdehyde (MDA) as the affected parameter. Briefly, the organs were homogenized 1:10 in PBS, and then, to 500 µl of homogenate from each tissue, we added 2 ml of the reaction mixture (TCA-TBA-HCl) at 15% w/v, 0.375 w/v, and 0.25 N, respectively. The mixture was boiled for 15 min, cooled in an ice bath for 10 min, and centrifuged at 4000 rpm for 10 min. Then, the supernatant was spectrophotometrically read at 532 nm against a reference blank. The concentration of MDA was calculated by using an extinction coefficient of 1.56 × 10^5^ M^-1^ cm^-1^. The results were expressed as nmol MDA/mg protein.

### Determination of oxidized proteins

This measurement was made through the quantification of the reactive carbonyl content according to the method of Levine et al.^18^. Each tissue was homogenized in PBS (1:10) followed by the addition of 500 μl of DNPH (10 mM in HCl 2 mM) to 200 µl of the tissue homogenate. The mixture was placed at room temperature in the dark for 1 h, and the generated hydrazones were precipitated with 500 μl of 20% TCA. Each sample was centrifuged three times at 9000 rpm for 10 min and each time, the suspension was washed with 1 ml of ethyl acetate-ethanol 1:1. The pellet was re-suspended in 1 ml of hydrochlorate guanidine 6 M, incubated at 37 °C for 15 min, and centrifuged at 9000 rpm for 10 min. For each sample, we concurrently followed the same procedure with a blank incubated with 500 µl of HCl 2 M without DNPH. The carbonyl content was spectrophotometrically registered in a range from 350 to 375 nm, and its concentration was calculated by using 22,000 M^-1^ cm^-1^ as the coefficient of molar absorbance. Results were expressed as nmol of CO•/mg protein.

### Nitric oxide determination

We prepared a homogenate (1:4) from each tissue by adding cold PBS. Then, 600 μl of the homogenate were centrifuged for 20 min at 4000 rpm and the supernatant was treated with the Griess reaction to determine the concentration of nitrites^19^. For this purpose, 300 μl of the Griess reactive plus 600 µl of distilled water were added to 100 μl of the obtained supernatant**.** The mixture was then measured at 540 nm. As a standard, we used NaNO_2_ 0.1 Mm in a range from 0.9 µmol to 10 μmol. The results were expressed as µmol of nitrite/g of tissue.

## Results

### Comet assay: Standard and with the FPG enzyme

**Figure 1:**
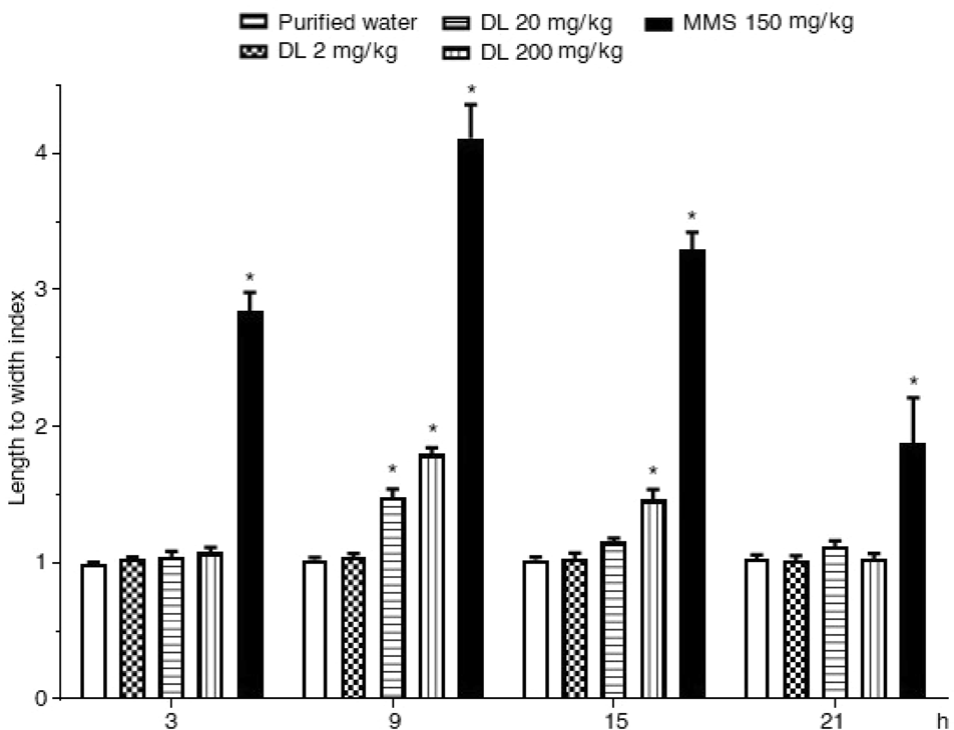
Mouse brain cells exposed to duloxetine (DL) and methyl metanesulfonate (MMS). Evaluation with the comet assay along 21 h after a single administration. Each bar represents the mean ± SEM of 5 mice per group. 100 nucleoids per animal. *Statistically significant difference with respect to control value. ANOVA and post hoc Student–Newman–Keuls tests (*p* ≤ 0.05).

The results of the comet tail length/nucleus diameter index obtained in the brain with the standard method are shown in Fig. 1. It was observed as a low and constant value along with the assay in the control group, a result that contrasts with the elevated DNA damage manifested by the exposure to MMS during the different examined time points. However, the effect was higher at 9 h post-exposure (four times over the control value) followed by a certain decrease, a behavior usually observed when the cells are exposed to a single administration. Concerning duloxetine, it was interesting to note a statistically significant damage increase also at 9 h post-exposure, indicating that this time was optimal to detect the DNA effect induced by the antidepressant. In the doses 20 and 200 mg/kg we found 50, and 80% increase over the control group, respectively, although the lower dose showed no genotoxic effect.

**Figure 2:**
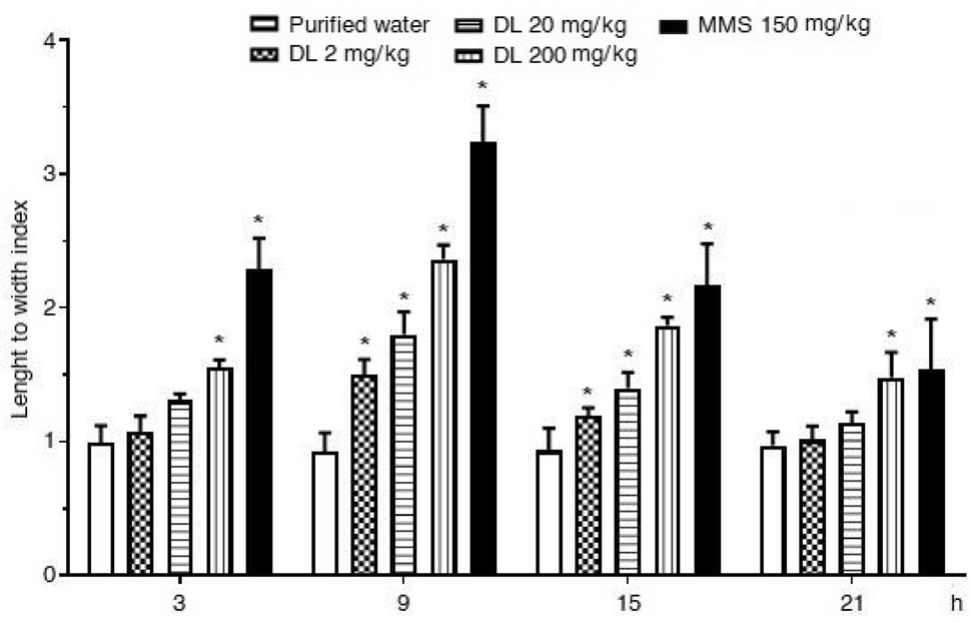
Mouse hepatic cells exposed to duloxetine (DL) and methyl metanesulfonate (MMS). Evaluation with the comet assay along 21 h after a single administration. Each bar represents the mean ± SEM of 5 mice per group. 100 nucleoids per animal. *Statistically significant difference with respect to control value. ANOVA and post hoc Student–Newman–Keuls tests (*p* ≤ 0.05).

As regards to the liver cells, the response observed in the negative and the positive control animals was similar to the described above (Fig. 2). Concerning duloxetine, the damage was more evident than in cerebral cells. The high dose induced a significant effect since the first observed data point, and the three tested doses were statistically significant from the control value at 9 and 15 h post-treatment. However, the highest damage was found after 9 h of exposure, similarly to those observed in brain cells. At this time, the increase of 2, 20, and 200 mg/kg of the drug was 50%, 80%, and 135% respectively, in comparison with the control level.

**Figure 3:**
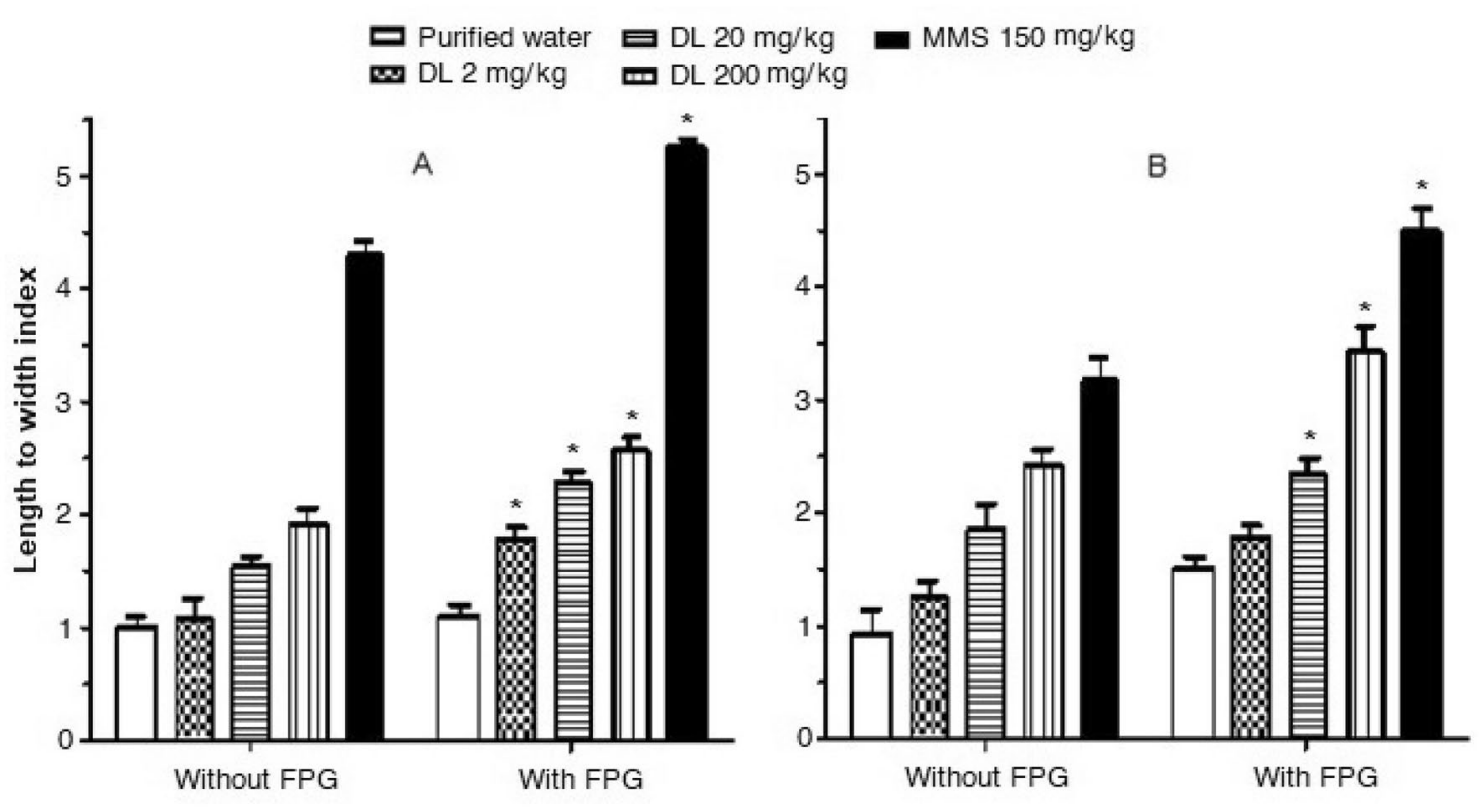
Mouse brain (**A**) and liver cells (**B**) treated with duloxetine (DL) and methyl metanesulfonate (MMS). Comet assay added or not with the enzyme formamido pyrimidine-DNA glycosilase (FPG). Results correspond to the effect of a single administration of each compounds evaluated at 9 h post-administration. Each bar correspons to the mean ± SEM obtained in 100 nucleoids per mouse, 5 mice per group. *Statistically significant difference with respect to the value without FPG. ANOVA and post hoc Student–Newman–Keuls tests (*p* ≤ 0.05).

To examine the influence of the DNA oxidation in our results we analyzed the effect of duloxetine in preparations added with FPG at 9 h after the drug’ exposure. Figure 3A shows the comparison of results obtained without and with the addition of the enzyme in brain cells, while Fig. 3B shows data obtained in hepatic cells. In the case of brain cells, we found that FPG provoked 59% comet increase when 20 mg/kg of duloxetine was administered, in comparison with the level determined without the enzyme; also, we observed a 77% DNA damage increase with the addition of 200 mg/kg duloxetine respect to the level determined with no enzyme added. Concerning hepatic cells, the effect was slightly stronger, 76% and 99% with 20 and 200 mg/kg over the control level, respectively. These results established the DNA oxidation effect of the antidepressant with the two high doses tested.

### Oxidative effect: Lipids, proteins, and nitric oxide

**Figure 4:**
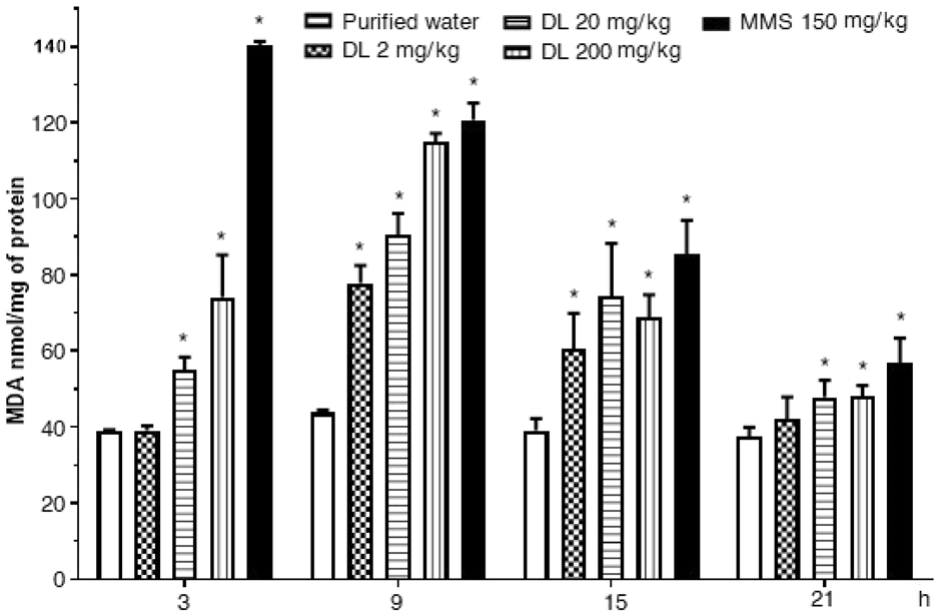
Effect of duloxetine (DL) and methyl metanesulfonate (MMS) on the content of malondialdehyde (MDA) in mouse brain cells. Each bar corresponds to the mean ± SEM obtained in 5 independent determinations made in triplicate. *Statistically significant difference with respect to control value. ANOVA and post hoc Student–Newman–Keuls tests (*p* ≤ 0.05).

Figure 4 shows the effect of duloxetine on brain lipoperoxidation. It was observed as a low and constant malondialdehyde level in the control group along with the assay, and a high malondialdehyde increase content induced by MMS, which showed a decreased curve with the assay. Concerning duloxetine, we determined a significant malondialdehyde increase at 3 h post-exposure with the two high doses; however, the more significant damage was expressed after 9 h of exposure, where even the low dose (2 mg/kg) produced a 75% damage increase in comparison with the control group. Even though the kinetic curve decreased after such schedule, duloxetine was also a lipid oxidative agent at 15 h and 21 h with the two high doses tested.

**Figure 5:**
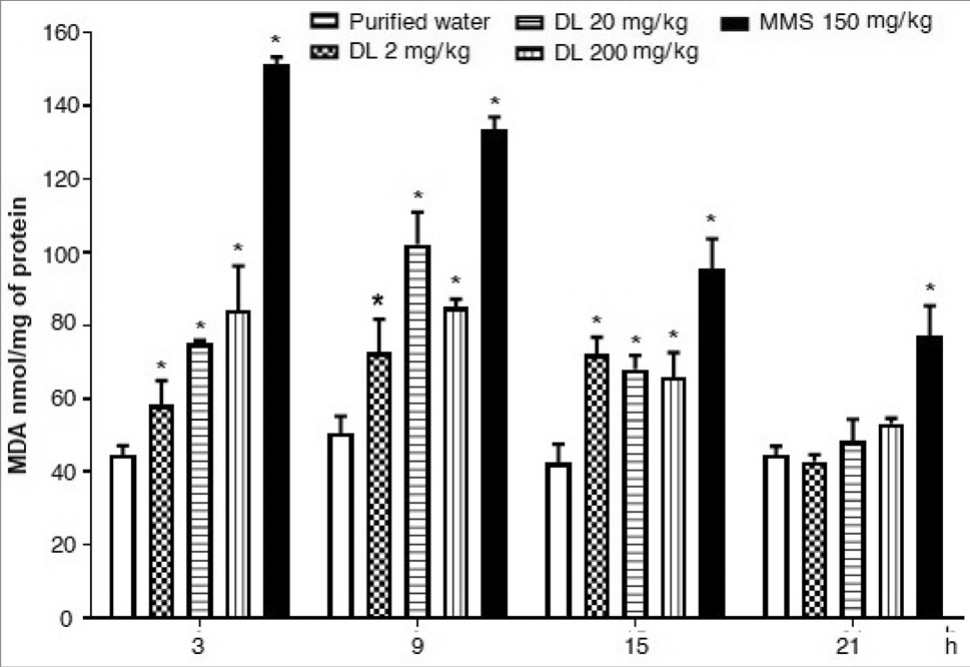
Effect of duloxetine (DL) and methyl metanesulfonate (MMS) on the content of malondialdehyde (MDA) in mouse hepatic cells. Each bar corresponds to the mean ± SEM obtained in 5 independent determinations made in triplicate. *Statistically significant difference with respect to control value. ANOVA and post hoc Student–Newman–Keuls tests (*p* ≤ 0.05).

A similar result was obtained concerning hepatic cells; however, in this case, the response of the drug was somewhat stronger (Fig. 5). While in the control and the MMS treated group, the malondialdehyde level showed similar behavior to those described in brain cells, the three doses of the antidepressant increased the lipid biomarker during the examined schedule except at 21 h. With respect to the control level, at 9 h post-exposure, the increase was 43, 102, and 168% with 2, 20, and 200 mg/kg, respectively.

**Figure 6:**
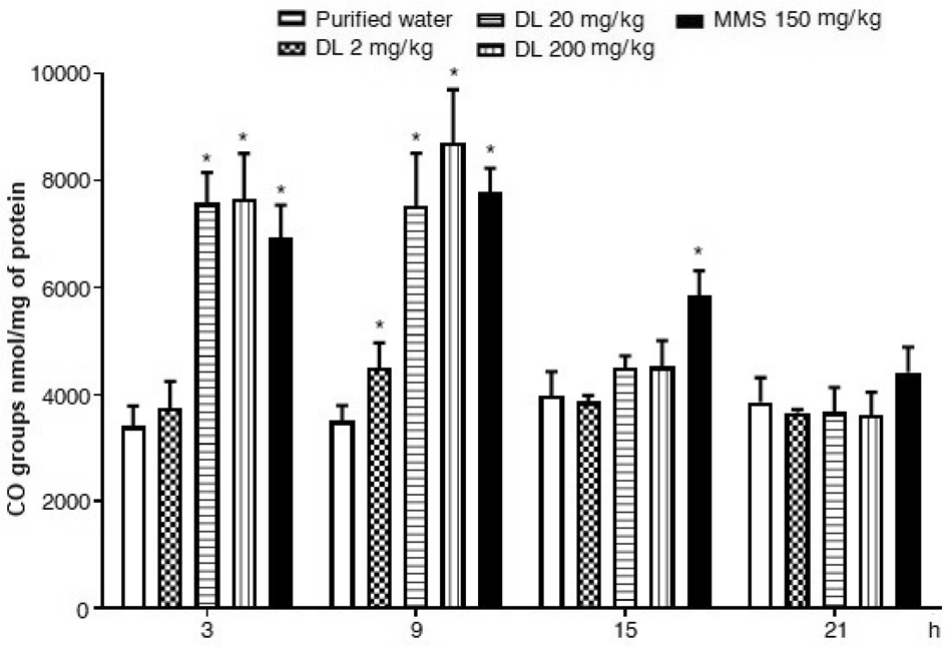
Effect of duloxetine (DL) and methyl metanesulfonate (MMS) on the content of oxidized carbonil groups (CO•) in mouse brain cells. Each bar corresponds to the mean ± SEM obtained in 5 independent determinations made in triplicate. *Statistically significant difference with respect to control value. ANOVA and post hoc Student–Newman–Keuls tests (*p* ≤ 0.05).

**Figure 7:**
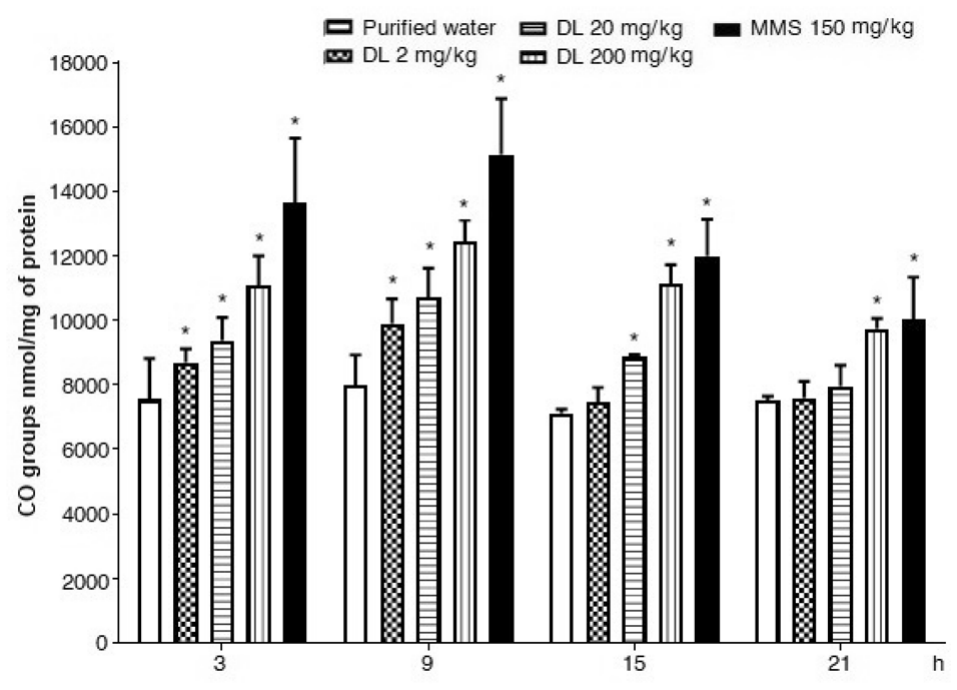
Effect of duloxetine (DL) and methyl metanesulfonate (MMS) on the content of oxidized carbonil groups (CO•) in mouse hepatic cells. Each bar corresponds to the mean ± SEM obtained in 5 independent determinations made in triplicate. *Statistically significant difference with respect to control value. ANOVA and post hoc Student–Newman–Keuls tests (*p* ≤ 0.05).

Results regarding oxidized proteins are presented in Figs. 6 and 7 respect to brain and hepatic cells, respectively. In the first figure, we observed a significant increase of oxidized carbonyls at 3 h and 9 h post-exposure, mainly induced with the two high doses tested, such increase was found in the range of the results observed with the selected positive mutagen. Results in liver cells (Fig. 7) showed that the three tested doses of duloxetine were statistically significant at 3 h and 9 h against the control level, a difference that was also present at 15 h with the two high doses, and at 21 h with the highest dose. At 9 h the elevation with respect to the untreated cells corresponded to 24%, 34%and 55% with 2, 20 and 200 mg/kg of duloxetine, respectively. At the same time, in the brain, the increase corresponded to 28%, 114% and 148% with 2, 20, and 200 mg/kg, respectively.

**Figure 8:**
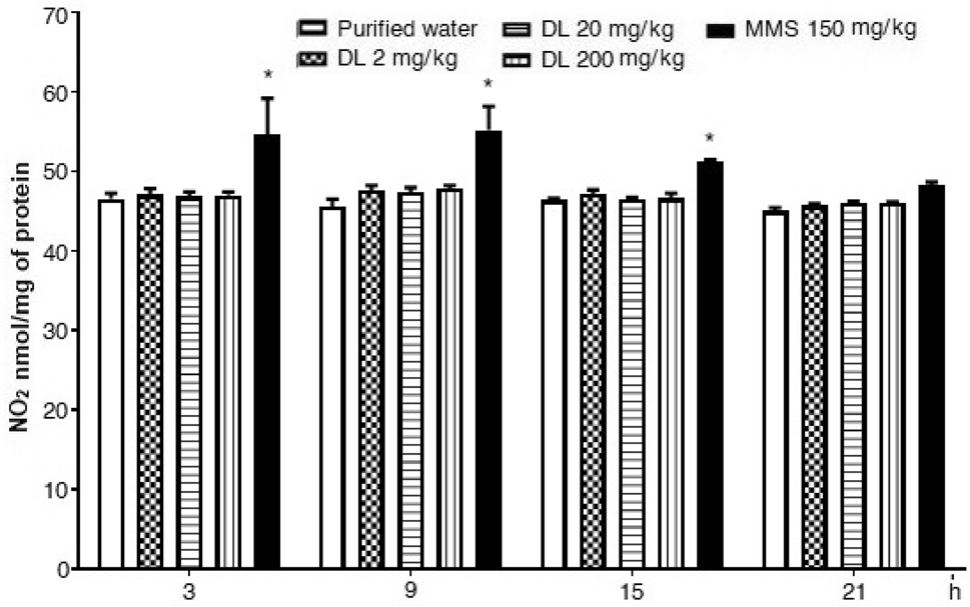
Effect of duloxetine (DL) and methyl metanesulfonate (MMS) on the content of  nitrites (NO_2_) in mouse brain cells. Each bar corresponds to the mean ± SEM obtained in 5 independent determinations made in triplicate. * Statistically significant difference with respect to control value. ANOVA and post hoc Student–Newman–Keuls tests (*p* ≤ 0.05).

**Figure 9:**
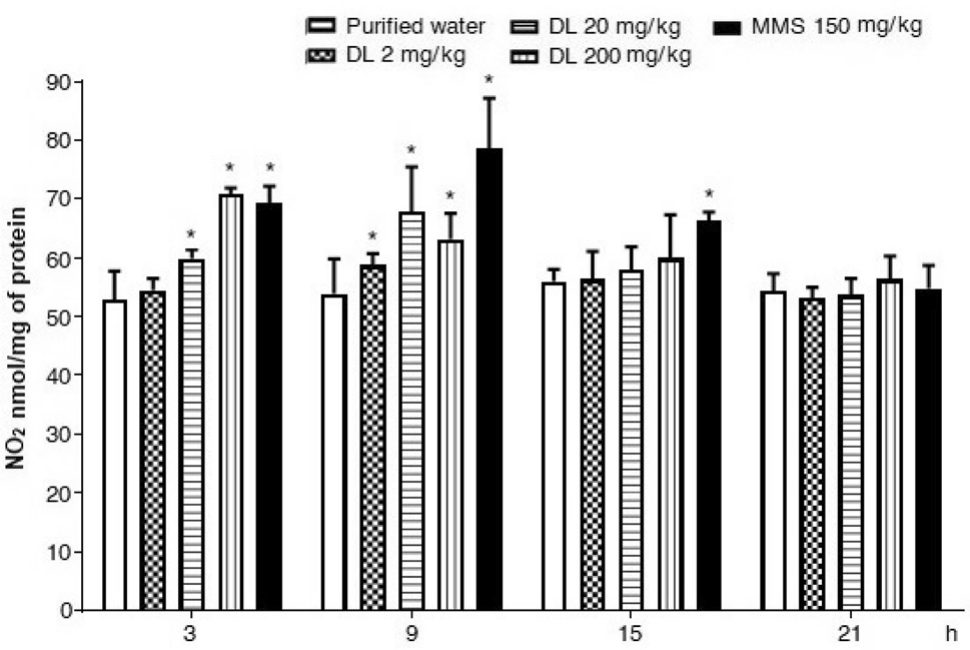
Effect of duloxetine (DL) and methyl metanosulfonate (MMS) on the content of  nitrites (NO_2_) in mouse hepatic cells. Each bar corresponds to the mean ± SEM obtained in 5 independent determinations made in triplicate. *Statistically significant difference with respect to control value. ANOVA and post hoc Student–Newman–Keuls test (*p* ≤ 0.05).

Finally, the results on oxidation products of nitrogen oxide are shown in Fig. 8 for the brain and in Fig. 9 for the liver. In brain cells, we observed no effect of duloxetine along with the whole assay, contrary to a moderate elevation of this parameter in hepatic cells. In comparison with the control value, the damage that was statistically significant at 3 and 9 h after the antidepressant exposure. Concerning untreated mice, at 9 h post-exposure, the increase with 2, 20 and 200 mg/kg of duloxetine was 9%, 26%, and 17% respectively.

## Discussion

There are approximately 350 million people worldwide with depression, a fact that supports the relevance of pharmacotherapy as a key role in the treatment of the disease^20^. Moreover, it is known that antidepressants may be used in short or long-term treatments, and, therefore, it is understandable the need for the safe use of such medications. In this context, it has been recognized that variations in the DNA molecule and its function, along with the effect of environmental influences are key factors that explain the development of numerous disorders such as single-gene diseases, chromosomal imbalances, epigenetics, cancer, and complex disorders^21^. In parallel with this knowledge, several assays to detect damage in DNA and chromosomes have been developed and widely applied, among several purposes, to examine the genotoxic effect of medications, including antidepressants, in in vitro and in vivo assays. One of these genotoxic methods is the single cell gel electrophoresis assay, usually known as comet assay, which is a versatile tool that has good sensitivity, adaptability, and reliability, and may be applied to most cells to measure DNA strand breaks, incomplete excision repair events, alkaline labile sites, and cross-linking events^22^.

In the present report, we initially applied the alkaline version of the comet assay to mouse liver cells administered duloxetine and found a high level of damaged DNA with the high dose at all examined times, moreover, more interesting was that the three tested doses were genotoxic in liver at 9 and 15 h of the assay. In the brain, however, the effect of the high dose was observed only at 9 and 15 h although with lower potency than in hepatic cells, and the intermediate dose (20 mg/kg) had a genotoxic effect only at 9 h post-administration. The stronger effect of duloxetine in liver cells was probably related to the chemical biotransformation process in such organ, where the drug is subjected to oxidation, methylation, and conjugation pathways, to the action of enzymes such as CYP2D6 and CYP1A1, and the formation of metabolites which include the glucuronide conjugate of 4-hydroxy duloxetine, and the sulfate conjugate of 5-hydroxy, 6-methoxy duloxetine^23^.

Our data suggest that the optimal time for duloxetine to induce DNA damage was at 9 h post-administration, a result which is congruent with the reported pharmacokinetic behavior of the drug, which has shown a high mean plasma concentration from 6 to 12 h and a t_1/2_ of 11.1 h^23,24^. Besides, the DNA damage time-course strategy applied to potential genotoxicants has been useful to examine the detoxification and DNA repair processes whether in cultivated cells or in in vivo assays. In our case, the break removal process started after 9 h post-treatment and its stronger effect reached the control level at 21 h with all tested doses in brain cells, and also with the two low doses in the case of liver cells. Our results show a time-curve with pre-damage, the maximum damage, and its decline to normality, which reflects the DNA damage potential and the repair process.

Concerning DNA oxidation, it is known that this may be caused by several oxidants that can produce base and sugar damage, strand breaks, clustered sites, and other lesions, and that these alterations are mainly repaired with the participation of DNA glycosylases^25^. In this context, the FPG protein is a DNA base excision repair enzyme that catalyzes the removal of oxidized purines, such as the mutagenic 7-hydro-8-oxoguanine lesion, by the activity of N-glycosylase^26^. At the location of oxidized DNA bases, additional DNA strand breaks occur which leads to DNA migration. This knowledge has been applied to the comet assay to detect the oxidative influence of numerous agents and, in the present study, to the oxidative potential of duloxetine. Interestingly, by using this approach we demonstrated the DNA oxidative effect of the drug, although with the two high doses tested, as shown by the significant elevation of DNA breaks when the FPG enzyme was incorporated to the comet assay, in comparison with the comet assay without such enzyme, an effect that was observed in both organs. In concordance with such findings, we also observed an elevated induction of lipoperoxidation by duloxetine, as well as oxidized proteins, and nitric oxide in liver cells, as well as of lipid peroxidation and oxidized proteins in brain cells, with a clear effect at 9 h post-administration in both organs; a time-point that coincides with that required to induce the highest DNA damage, as shown with the comet assay.

Oxidative stress is the imbalance in the redox characteristics of some cellular environments, which can be the result of exposure to damaging agents or to the limited capabilities of endogenous antioxidant systems^27,28^. In our present study, we found oxidation in all evaluated molecules, suggesting a higher damaging potential of duloxetine than previously reported.

Oxidation of DNA components is the major source of induced DNA damages leading to several types of modifications including nucleotide oxidation, strand breakage, loss of bases, and adduct formation. The HO• radical can react with purine and pyrimidine bases and deoxyribose backbone generating products such as the 8oxodG^29^. Damaged DNA replication may lead to gene mutation, which in turn may give rise to altered proteins. Besides, mutations that affect an oncogene, a tumor suppressor gene, or a gene that controls the cell cycle can generate a clonal cell population with a distinct advantage in proliferation^30^.

Lipid peroxidation is a process under which oxidants attack lipids containing carbon–carbon double bonds, especially polyunsaturated fatty acids. One of the most prevalent ROS that can affect the lipids is the hydroxyl radical, a small, highly mobile, water soluble and highly reactive species of activated oxygen, this radical, can cause oxidative damage to cells because non-specifically attack biomolecules close to its site of generation^31^. In general, when oxidant compounds target lipids, they can initiate the lipid peroxidation process, a chain reaction that produces multiple breakdown molecules, such as MDA and 4-hydroxy-nonenal. Among various substrates, proteins and DNA are susceptible to modifications caused by these aldehydes. Besides, the adducts play a critical role in multiple cellular processes and can participate in secondary deleterious reactions, by proting intramolecular or intermolecular protein/DNA crosslinking that may induce profound alterations in the biochemical properties of biomolecules^31,32^.

As proteins are highly abundant and react rapidly with many oxidants, they are highly susceptible and major targets to oxidative damage. Thus, oxidant alteration in most biological systems is likely to be skewed toward proteins, although other factors play an important role, including localization of the generating system relative to the target, membrane barriers, binding of the oxidant system to a target, and the occurrence of secondary reactions^33^. A number of radicals, two-electron oxidants, and metal-oxo complexes may modify proteins also reactions of secondary products, such as aldehydes, quinones and dehydroalanine are a further source of modifications^34,35^. Carbonyl groups can be generated by different mechanisms and, therefore, their concentration is commonly higher than other biomarkers^36^. Due to these characteristics, the measure of carbonyl levels is the most used marker of oxidative protein damage. Protein carbonyls can be formed by the oxidative cleavage of protein backbone, oxidative deamination of lysine and glutamic acid, or by binding of aldehydic lipid oxidation products to lysine, cysteine, and histidine residues. Also, the reaction between lysine and arginine residues with carbohydrates result in advanced glycation end products^37^.

The free radical nitric oxide (NO^•^) exerts biological effects through direct and reversible interactions with specific targets, such as soluble guanylate cyclase, or through the generation of secondary species, many of which can oxidize, nitrosate or nitrate biomolecules^38,39^. The species formed downstream by NO• include nitrogen dioxide, dinitrogen trioxide, nitroxyl, and peroxynitrite, as well as hydroxyl and carbonate anion radicals^37^. Many of these products are reactive and yield further products. Peroxynitrite for example, generate nitrites, nitrates, hydroxyl radicals, and carbonate anion radicals^37,38^. The preferential targets of oxide nitric derived oxidants in biological systems are located in close proximity and determined by a combination of factors, including target concentration, compartmentalization, and membrane permeability. Moreover, some of these derived oxidants are good one-electron oxidants that start oxygen-dependent chain reactions in both aqueous and lipid compartments, which may amplify the effects^38^.

The above described characteristics of the examined biomarkers demonstrate their relevance at the molecular and cellular level, moreover because they can interact among them to increase their damaging potential, and because all reports point to the fact that their alterations are reflected in human disease, such as aging, inflammation, cancer, and particular damage in the nervous, cardiovascular, immune, metabolic, endocrine, renal, and respiratory systems^30,32,37,40,41^. Therefore, our findings clearly suggest the importance to confirm or modulate the described effect of duloxetine. Is in this field, that experimental sub-chronic or chronic research can be carried out, as well as the appropriate monitoring of patients under long-term treatment.

Our observed molecular oxidation could be attributed to the participation of duloxetine epoxide during the formation of dihydrodiol-duloxetine and 5 hydroxy or 6 hydroxy duloxetine during duloxetine metabolism^42^, or because of the bioactivation of the naphthyl ring to generate quinones or epoxides, or related to the thiophene ring that may be bioactivated to generate epoxides, ring-opening or S-oxidation products^43^. In this respect, it is known that epoxides may give rise to point mutations, deletions, chromosomal aberrations, gene conversion, crossing over, cancer, and virus induction. Moreover, the release of free radicals or ROS by epoxide metabolites have also been suggested to participate in the hepatotoxic damage induced by duloxetine^44^.

Our oxidative findings are congruent with the report by Czarny et al.^45^ on depressed patients under treatment, because these authors determined a higher level of DNA breaks, alkali-labile sites, and oxidative DNA damage in the patients in comparison with normal individuals, and concluded that the observed lesions may be accumulated by impairment of repair systems; moreover, the authors also refer to previous reports showing increased levels of 8-oxo-G in urine, serum, or peripheral blood of patients. However, the oxidation of lipids, proteins, and nitric oxide by duloxetine had not been reported before in our present experimental conditions, and, therefore, the findings suggest the need to extend studies on the matter to ratify the observations or to modulate them. Besides, the oxidative effect by the antidepressant seems an interesting investigative and theoretical theme in light of the published controversial data. Various authors have reported neuroprotection exerted by duloxetine against oxidation, for example, by decreasing the level of dismutase and glutathione peroxidase in rats, by the lowering of intracellular rat neuron ROS production, antagonizing rotenone-induced overproduction of ROS and cell death in human neuroblastoma cells, or by increasing antioxidative capacity in patients under antidepressant treatment^45–49^.

In conclusion, we demonstrated DNA damage induced by duloxetine by means of a time-kinetic study, particularly in liver tissue where the increase was found even with the low tested dose, which corresponds to the high therapeutic range recommended for depressed patients. In our assay, we were able to follow the behavior curve when a single administration of the drug was administered, and found the highest DNA damage at 9 h post-administration, followed by a repair up to 21 h. The basis of our duloxetine damaging findings are probably connected with the oxidation determined in DNA, lipids, proteins, and nitric oxide. Therefore, our results strongly suggest the pertinence to extend the research on the potential toxic effect of duloxetine, as well as to be cautious with the long-term drug prescription.
